# Long-Term Survival and Local Relapse Following Surgery Without Radiotherapy for Locally Advanced Upper Rectal Cancer: An International Multi-Institutional Study

**DOI:** 10.1097/MD.0000000000002990

**Published:** 2016-06-03

**Authors:** Jun Seok Park, Yoshiharu Sakai, NG Siu Man Simon, Wai Lun Law, Hyeong Rok Kim, Jae Hwan Oh, Hester Cheung Yui Shan, Sang Gyu Kwak, Gyu-Seog Choi

**Affiliations:** From the Colorectal Cancer Center (JSP, G-SC), Kyungpook National University Medical Center, School of Medicine, Kyungpook National University, Daegu, Korea; Department of Surgery (YS), Kyoto University Hospital, Kyoto, Japan; Department of Surgery (NGSMS), The Chinese University of Hong Kong, Sha Tin, Hong Kong; Division of Colorectal Surgery (WLL), The University of Hong Kong, Pok Fu Lam, Hong Kong; Department of Surgery (HRK), Chonnam National University Hwasun Hospital and Medical School, Gwangju, Korea; Center for Colorectal Cancer (JHO), National Cancer Center Hospital, Goyang city, Korea; Department of surgery (HCYS), Pamela Youde Nethersole Eastern Hospital, Chai Wan, Hong Kong; and Department of Medical Statistics (SGK), School of Medicine, Catholic University of Daegu, Korea.

## Abstract

Controversy remains regarding whether preoperative chemoradiation protocol should be applied uniformly to all rectal cancer patients regardless of tumor height. This pooled analysis was designed to evaluate whether preoperative chemoradiation can be safely omitted in higher rectal cancer.

An international consortium of 7 institutions was established. A review of the database that was collected from January 2004 to May 2008 identified a series of 2102 patients with stage II/III rectal or sigmoid cancer (control arm) without concurrent chemoradiation. Data regarding patient demographics, recurrence pattern, and oncological outcomes were analyzed. The primary end point was the 5-year local recurrence rate.

The local relapse rate of the sigmoid colon cancer (SC) and upper rectal cancer (UR) cohorts was significantly lower than that of the mid/low rectal cancer group (M-LR), with 5-year estimates of 2.5% for the SC group, 3.5% for the UR group, and 11.1% for the M-LR group, respectively. A multivariate analysis showed that tumor depth, nodal metastasis, venous invasion, and lower tumor level were strongly associated with local recurrence. The cumulative incidence rate of local failure was 90.6%, 92.5%, and 94.4% for tumors located within 5, 7, and 9 cm from the anal verge, respectively.

Routine use of preoperative chemoradiation for stage II/III rectal tumors located more than 8 to 9 cm above the anal verge would be excessive. The integration of a more individualized approach focused on systemic control is warranted to improve survival in patients with upper rectal cancer.

## Abstract 2

Supplemental Digital Content is available in the text

## INTRODUCTION

The last 2 decades have witnessed the development of surgical standards, including total mesorectal excision (TME), which has been shown to decrease locoregional recurrence (LR) significantly.^1,2^ Several large clinical trials have demonstrated the superiority of neoadjuvant radiotherapy with chemotherapy over surgery alone. Preoperative chemoradiotherapy (CRT) is also associated with higher sphincter-saving procedures and improved local control.^3–8^ For patients who are selected to receive CRT, the expected benefits and disadvantages have to be well balanced, because pelvic radiotherapy may be provided at the expense of increased late morbidity and toxicity. Preoperative CRT also has major financial implications for health care providers.

There is no agreement regarding the cutoff height from the anal verge of the rectum at which patients would not benefit from preoperative CRT. According to the guideline of the National Comprehensive Cancer Network, preoperative CRT is recommended for all rectal cancers of clinical stages II and III, regardless of the distance between the tumor and the anal verge.^9^ Previous major rectal cancer trials that assessed the efficacy of CRT have defined rectal cancer as any tumor located <15 or 16 cm from the anal verge, and included upper rectal cancer.^3–6,10–13^ However, routine administration of CRT before proper surgery has been questioned particularly for those with upper rectal cancer. Several studies showed that higher rectal cancers behave like colon cancers regarding recurrence pattern and prognosis, and not like rectal cancer.^14,15^

Because of the variation in protocols regarding preoperative CRT and the paucity of literature on this subject, we aimed to estimate the impact of omitting radiotherapy in upper rectal cancer. Therefore, we analyzed the long-term outcomes of upper rectal cancer and compared them with those of sigmoid and low rectal cancer in a cohort of patients treated with a non-preoperative CRT protocol. In addition, this study was conducted to evaluate whether it is possible to establish an individualized anatomical landmark for preoperative CRT by assessing the difference in oncological outcomes according to the peritoneal reflection (intraperitoneal vs extraperitoneal).

## PATIENTS AND METHODS

### Patients Cohort

An international consortium of 7 institutions, each of which is a tertiary cancer center, was established. Consecutive cases of operations performed between January 2004 and May 2008 comprised the data set for this analysis. Eligibility criteria included biopsy-proven adenocarcinoma, with the inferior margin located within 30 cm of the anal verge. A prospectively maintained administrative database or the hospital records of all 2962 patients with tumor stage II or III rectal and sigmoid colon cancer were reviewed. Eight hundred sixty-four patients were excluded for the following reasons: age >80 years (n = 156), synchronous or previous history of other malignancies (n = 148), emergent cases (n = 82), loss to follow-up within 6 months (n = 44), history of neoadjuvant therapy (n = 208) or adjuvant radiotherapy (n = 144), and familial adenomatous polyposis or hereditary nonpolyposis colorectal carcinoma (n = 78). Thus, the study population consisted of 2102 patients. Each surgeon who participated in this study provided specified perioperative and pathological data using a common menu-driven database file that incorporated precise coding instructions. The study was approved by the institutional review board of Kyungppk National University Medical Center.

### Tumor Location

The primary aims of the current analysis were to describe and compare the 5-year LR rate of tumors according to their location. Subgroups of particular interest were the intraperitoneal subset of patients compared with those with extraperitoneal rectal cancer. The tumor height was defined as the distance between the tumor caudal margin and the anal verge, and was measured by rigid sigmoidoscopy or colonoscopy. Tumors located 15.1 to 30 cm, 10.0 to 15 cm, and 0 to 9.9 cm from the anal verge were classified as sigmoid cancer (SC group, control arm), upper rectal cancer (UR group, investigational arm), and cancer of the mid- or lower-third rectum (M-LR group, control arm), respectively. In subgroup analysis, the final discrimination for categorization between intraperitoneal and extraperitoneal lesions was done intraoperatively by identification of the anatomical differences by the surgeon. Generally, rectal cancer was classified as extraperitoneal when the cranial margin of the tumor was located below the peritoneal reflection.

### Treatment Scheme

The surgical technique used at each institution was standardized in terms of the tumor-specific mesorectal excision principle, as described previously.^16–18^ Although postoperative care varied slightly across institutions, most surgeons had established a similar protocol for postoperative treatment. Postoperative chemotherapy was administered according to local unit policy, predominantly Mayo regimen or oral capecitabine. Adjuvant chemotherapy was administrated to patients with stage III disease and to those with stage II disease with poor clinicopathological features.

Recurrences were confirmed by pathology or conventional imaging techniques. LR was defined as evidence of recurrent disease within the pelvis, including recurrence at the site of anastomosis and perineum, whereas distant metastasis was defined as any other recurrence. The local recurrence was further subdivided into lateral pelvic type and central type. Lateral pelvic recurrence was defined as recurrence in the muscle (piriformis, elevator), soft tissue of the pelvic sidewall, lymph nodes along the iliac vessels, lateral pelvic nerve plexus, and lateral bony pelvis. Central pelvic recurrence was defined as recurrence in the perianastomotic area, posterior tumor bed (presacral), and anterior pelvic organs (bladder, prostate, vagina, etc.).

### Sample Size Calculation and Statistical Analysis

All statistical analyses were performed using the IBM SPSS Statistics software package for Windows (IBM SPSS Statistics version 20.0, New York, NY). Quantitative variables were expressed as the value of mean (standard deviation) and qualitative variables were expressed as the value of frequency (proportion). Between-group differences for total set and subgroup set were compared using one-way ANOVA or two-sample *t* test for quantitative variables with normality and chi-square test for qualitative variables, as appropriate. Probability of overall survival (OS) and probability of disease-free survival (DFS) were estimated using the Kaplan-Meier method. The log-rank test was used to compare survival rate between groups. Minimum length of follow-up is 5 years. Selected variables showing statistical significance in a univariate analysis were included as covariates in multivaiate analysis, binary multiple logistic regression. For the selection of optimal covariates in multivariate analysis, the forward conditional likelihood method was used. With the result of binary multiple logistic regression, the values of odds ratio (OR), 95% confidence interval of OR, and *P* value were presented for statistically significant variables. Propensity score matching method used in Figure 2 are presented as a supplementary file 1. Differences were considered significant if *P* < 0.05.

## RESULTS

Patient characteristics are listed in Table 1. There were no differences in most baseline variables but oxaliplatin-based chemoregimens (i.e., FOLFOX and FOLFIRI) were administered significantly more frequently in the SC group than in the UR and M-LR groups (*P* < .001). The overall R0 resection rate was 98.4%.

**TABLE 1:**
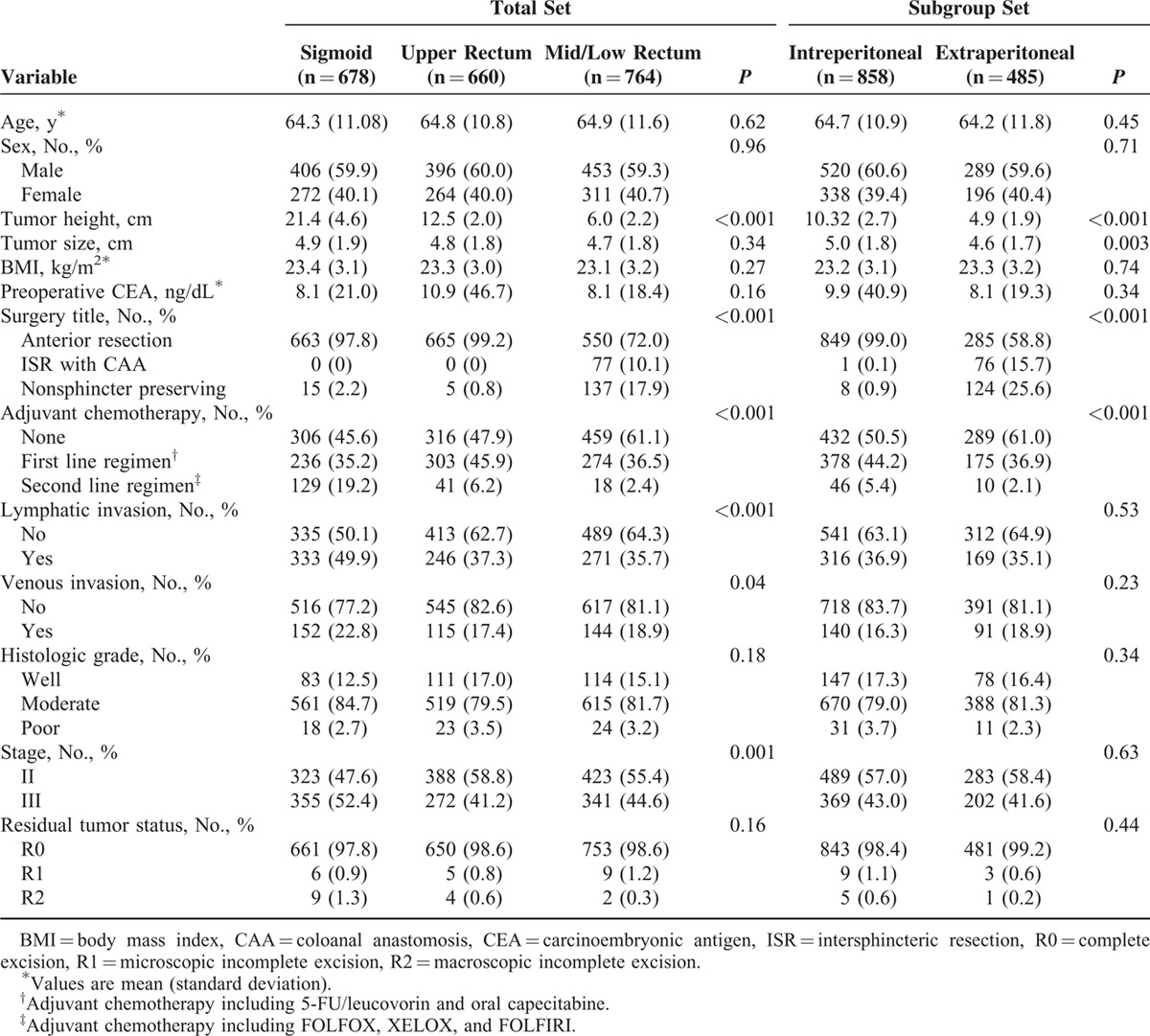
Patient and Tumor Characteristics

### Pattern of First Failure

The median follow-up period was 79.0 months (interquartile range, 62.1–97.6 months), and, in total, 540 (25.7%) patients developed disease recurrence in 5 years after primary surgery. The distribution of the first-recurrence events according to tumor location is presented as supplementary Table 1. The isolated local recurrence rate was 1.8% for the SC group, 2.7% for the UR group, and 10.8% for the LR group (*P* < 0.001). Regarding local relapse, lateral pelvic recurrence was prominent in the M-LR group, whereas relapse at the central pelvis occurred more often in the SC and UR groups. Moreover, there were some differences between the 3 groups regarding the pattern of distant recurrence. The most common hematogenous recurrence site was the liver in the SC group and UR group, whereas it was the lung in the M-LR group.

### Survival Outcomes

**FIGURE 1:**
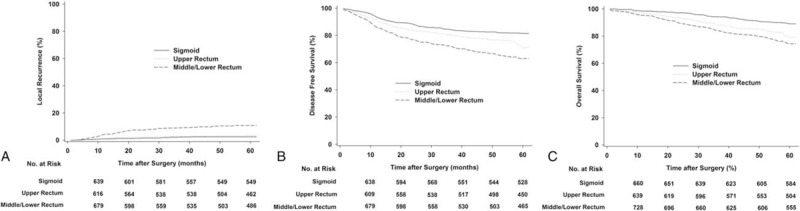
Estimated local recurrence (A), disease-free survival (B), and overall survival (C) for all patients according to tumor location.

The overall 5-year LR, DFS, and OS were dependent on tumor location (Table 2). The local relapse rate of the SC and UR cohorts was significantly lower than that of the M-LR. There were statistically significant differences in hazard ratios (HRs) for LR comparison among all patients, favoring the SC and UR groups (SC vs UR: HR, 0.583; 95% CI, 0.172–1.972; *P* = 0.39; UR vs M-LR: HR, 0.819; 95% CI, 0.185–3.620; *P* < 0.001; and M-LR vs SC: HR, 5.07; 95% CI, 2.87–8.98; *P* < 0.001). Regarding DFS and OS, a Kaplan–Meier analysis and post hoc tests showed that the curve of the UR group lay between that of the SC and M-LR groups (Figure 1). Similar results were also obtained for the LR, DFS, and OS rates stratified by stage. The 5-year DFS rate for the stage III UR group compared with the stage III SC group was 65.2% vs 78.3% (HR, 1.759; 95% CI, 1.298–2384; *P* < 0.001). Subsequently, we specifically studied the relapse rates according to T and N stage to identify high-risk subpopulations. The patients with upper rectal cancer were divided into 4 groups according to TN substaging (supplementary Table 2). Among the patients in the UR group, the T3N0, T3N1–2, T4N0, and T4N1–2 subgroups exhibited a 5-year LR of 1.2%, 5.7%, 5.6%, and 17.2%, respectively.

**TABLE 2:**
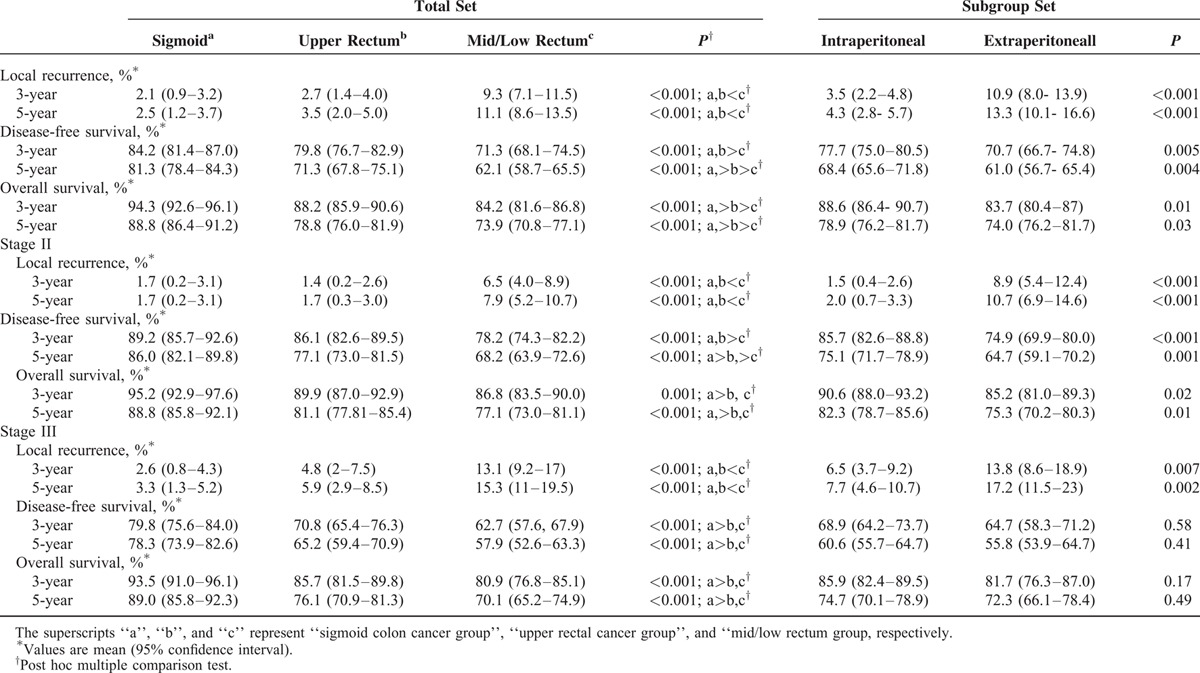
Oncologic Outcomes for the Study Population

### Multivariate Analysis and Estimated Cumulative Incidence of Local Recurrences

**FIGURE 2:**
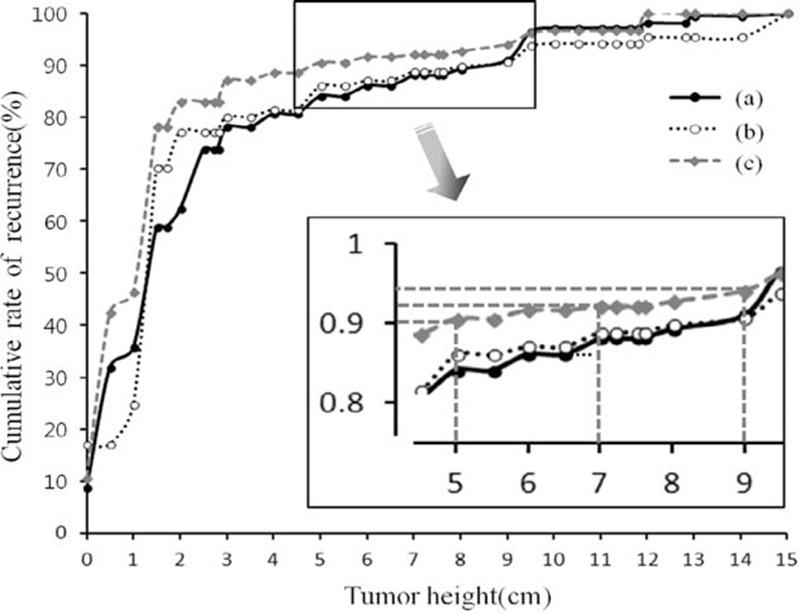
Predicted model for cumulative local relapse rate according to the tumor height: A, curve was drawn using raw data; B, curve was drawn after removing effects of 3 variables, venous invasion, mesenteric LN metastasis, and depth of tumor that were statistically significant in multivariate analysis; at last, C, curve was drawn after removing effects of all variables.

The results of the univariate analysis of risk factors for local relapse are given in Table 3. The multivariate analysis of local recurrence selected the following 4 independent risk factors: venous invasion, involvement of lymph nodes, T4 depth of the tumor, and lower tumor location. Using the confounding factor obtained from the multivariate analysis, a statistical model was created to estimate each cumulative incidence rate of LR according to tumor height. As we control potential confounding variables, the cumulative incidence rate of LR was 90.6%, 92.5%, and 94.4% for tumors located within 5, 7, and 9 cm of the anal verge, respectively (Figure 2).

**TABLE 3:**
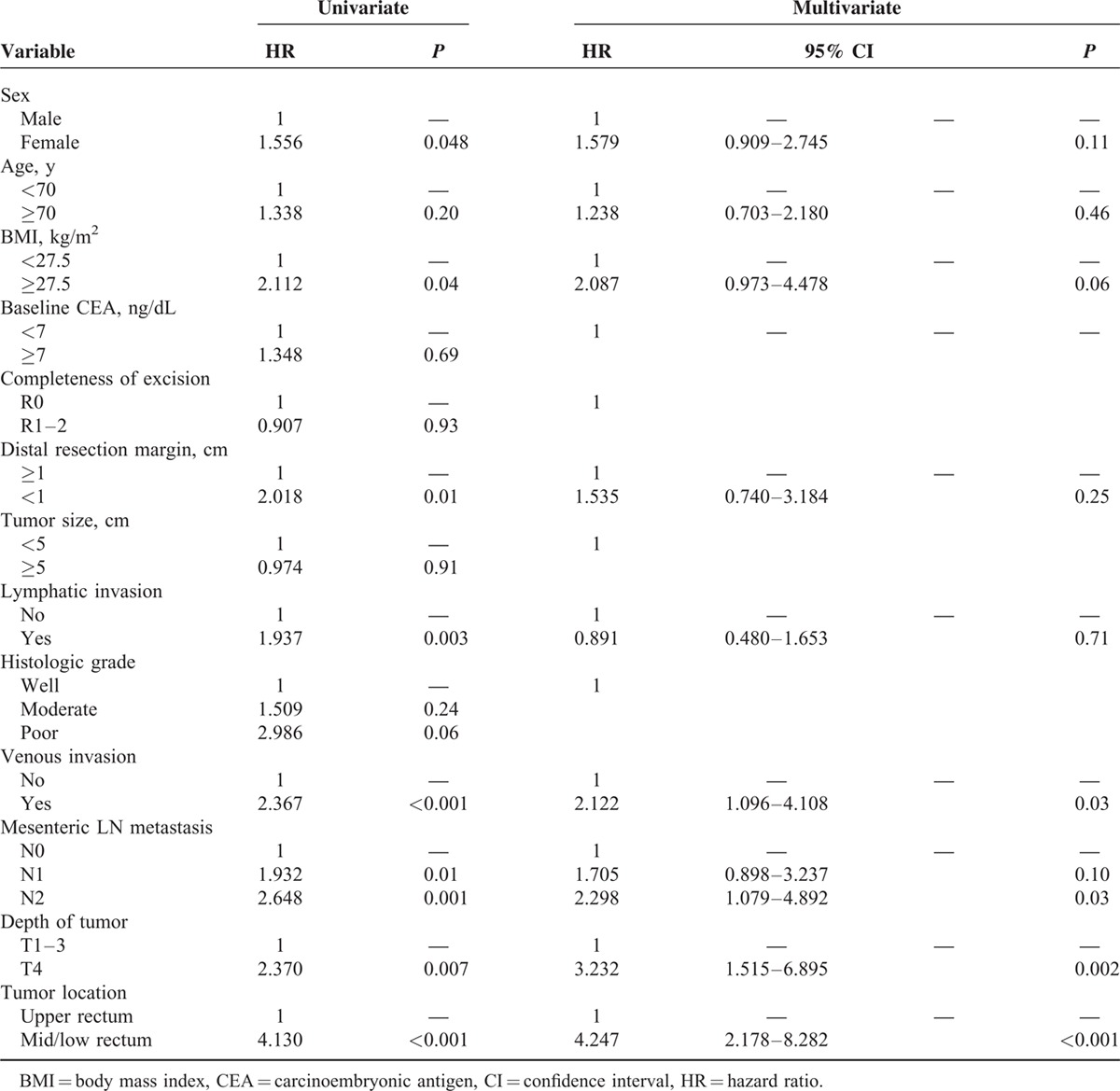
Multivariate Cox Regression Analysis of Local Recurrence Risk Among Patients With Rectal Cancer

### Subgroup Analysis: Intraperitoneal Versus Extraperitoneal Rectal Cancer

**FIGURE 3:**
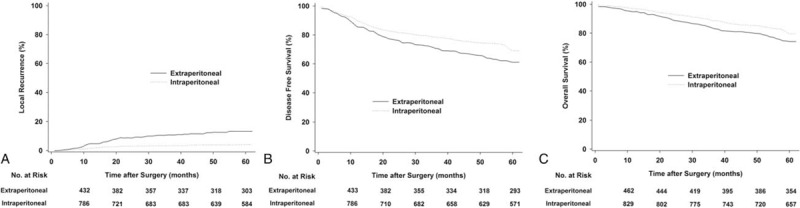
Estimated local recurrence (A), disease-free survival (B), and overall survival (C) for subgroup analysis: intraperitoneal versus extraperitoneal tumor.

After excluding sigmoid colon cancer, a subgroup analysis was performed to compare oncological outcome between tumor with extraperitoneal and those with intraperitoneal rectal cancers; the cancers were categorized according to peritoneal reflection. The most common hematogenous recurrence site in the intraperitoneal rectal group was the liver, whereas it was the lung in the extraperitoneal group. Significant differences in LR and DFS rates were found between intraperitoneal versus extraperitoneal tumors (*P* < 0.05) (Figure 3).

## DISCUSSION

Compared with the Western countries, preoperative chemoradiation has been introduced relatively late in the majority region of Asia. Indeed, preoperative CRT has been gradually integrated into the protocol of locally advanced rectal cancer management since the mid-2000s at these 7 institutions. During the study period, their use was limited to highly selected patients (advanced T4 tumor, clinical involvement of resection margin), thus providing a unique opportunity to compare sigmoid colon and upper rectal cancer with low rectal cancer directly, without confounding from radiation therapy. Without pelvic radiotherapy, LR rates of 1.7% and 5.9% were achievable in pathologically proved stage II and stage III cancer of the UR group, respectively. The statistical model for cumulative incidence rate of local relapse displayed a stronger linear association between tumor height and events. Higher lesions, which were located >9 cm from the anal verge, accounted for only 5.6% of all local failure. Taken together, these results lead us to believe that the absolute benefit of preoperative CRT in locally advanced upper rectal cancer may be small, if present.

The majority of surgeons consider that high rectal cancers should be treated according to the colon cancer guidelines. This treatment strategy is based on the hypothesis that cancers located in the upper third of the rectum behave more like colon cancer from a technical and anatomical perspective. Although TME has become a standard of care for rectal cancer, several conditions inevitably escape the TME field, for example, higher involvement rate of circumferential radial margin, potential microscopic tumor cell infiltration in pelvic soft tissue, and lateral spread of pelvic lymph nodes.^3,19^ Therefore, preoperative or postoperative adjuvant treatment has been added to TME. In contrast to low and middle tumors, which are constrained by a restrictive pelvis, upper rectal cancers are not bound by similar physical limitations. Several studies have shown that upper rectal cancer should be treated using the same technique (partial mesorectal excision) as sigmoid colon cancer, whereas TME should be performed for middle and low rectal cancers.^17,20^ In addition, one could argue that the lymphatic spread pattern of upper rectal cancer is different from that of mid or low rectal tumors. Lymphatic drainage from the upper rectum proceeds mostly along the superior rectal artery to the inferior mesenteric vessels. Conversely, the lower rectum has an additional portion that may drain laterally through the internal iliac system to the lateral pelvic sidewall.^21,22^ The incidence of lateral lymph node metastasis ranges from 8.6% to 27%, and such nodes are a major cause of local recurrence, even after preoperative CMT combined with TME.^21,23–25^ In our series, the lateral pelvic recurrence rate was 1.2% in patients with upper rectal cancer, and 7.3% in those with middle and lower rectal cancer. Our findings are consistent with those of prior studies, as they indicate that the incidence of lateral lymph node metastasis increases with decreasing distance of the tumor from the anal verge.^21,26^

There is some discrepancy in the impact of pelvic radiotherapy in upper rectal cancer between the historical randomized trials. Recently, the German Rectal Cancer Study Group (CAO/ARO/AIO-94) reported long-term results of that trial after a median follow-up of 134 months.^5^ The study population was composed of patients with T3/4 tumors that were diagnosed using endorectal ultrasound and computed tomography, 15% of whom had upper rectal lesions (>10 cm from the anal verge). The authors found a 10-year local recurrence of 4.3% in upper rectal tumors with preoperative CMT compared with 10.4% in the nonirradiated group. A Medical Research Council study also reported a reduction of the local recurrence rate after radiotherapy in patients with upper-third rectal cancer (1.2% with preoperative CRT vs 6.2% after selective postoperative CRT).^6^ In contrast, 2 other clinical trials reported results that support the hypothesis that preoperative CRT does not confer much additional benefit for upper rectal cancer. The subgroup analysis of the Swedish Rectal Trial and the Dutch TME trial proved the clinical efficacy of neoadjuvant CRT for mid and low rectal tumors; however, its effect on upper rectal lesions was not significant.^4,27^ None of the aforementioned trials focused on, or were powered, to evaluate the impact of radiation in the subset of patients with upper rectal tumors. In fact, the German Rectal Cancer Study Group performed subgroup analyses for upper lesions, included sample sizes between 45 and 85 patients according to treatment arms, and may have been underpowered.

An unexpected finding of the present study was that patients with sigmoid cancer showed a trend of improvement of the estimated disease-free survival and overall survival compared with cancers of the upper-third rectum. We postulate that these differences may have been caused by the adjuvant chemotherapy setting and the heterogenetic biological features of upper rectal cancer. During the study period, and in the setting of colon cancer, advanced drugs (including oxaliplatin, irinotecan, and the target agents) were established as a new standard regimen for stage III colon cancer. In contrast, the identification of optimal adjuvant chemotherapy protocols for rectal cancer has been complicated by the fact that most large clinical studies were conducted based on a neoadjuvant CRT setting.^28,29^ Indeed, in our series, a more aggressive chemoregimen was administered to the colon cancer cohort approximately 3 times more often than it was to the upper rectal cancer group.

The decision to administer radiation therapy based solely on numerical tumor height involves anatomical pitfalls. As conventionally described, the peritoneum runs obliquely and downward from the posterior reflection to the anterior reflection.^30^ Rectal cancers located 6 to 12 cm from the anal verge can be intraperitoneal tumors that would be too mobile to be reliably targeted with radiation or extraperitoneal tumors that should be amenable to radiotherapy.^30–32^ Therefore, some surgeons propose that peritonealization might be the individualized landmark for the selection of the upper boundary of the radiation field. In our subgroup analysis, the overall 5-year local recurrence rate in the intraperitoneal and extraperitoneal groups was 4.2% and 13.3%, respectively. More important, blood-borne metastases or disseminated disease predominated among intraperitoneal rectal tumors, whereas local failure was more frequent among tumors involving the area below the peritoneal reflection. In future studies, the authors plan to elucidate whether the peritoneal reflection is an individualized landmark that facilitates the selection of patients for preoperative CRT.

There were several limitations to this study. First, because of its retrospective nature, the impact of preoperative CRT on rectal cancer was not addressed adequately in this series. We were not able to compare protocol-for-protocol long-term outcomes between patients who did and those who did not undergo radiation therapy in addition to surgery, because only a very few patients (<4%) with upper rectal cancer received radiotherapy during the study period. Second, our analysis was performed using only Asian populations from 3 countries. Therefore, further validation using cohorts with different ethnicities or geographic locations would be recommended.

In conclusion, this study provided further evidence that omission of preoperative CRT may not represent undertreatment in locally advanced upper rectal cancer. The systemic control of micrometastasis and accurate pretreatment identification of high-risk T4 tumors may represent the next breakthrough in the management paradigm of upper rectal cancer. The definition in greater detail of the oncological implications of peritoneal reflection may allow the application of patient-tailored treatment strategies to advanced rectal cancer in the future.

## Supplementary Material

Supplemental Digital Content
