# Supplementary material for: Long-Term Survival and Local Relapse Following Surgery Without Radiotherapy for Locally Advanced Upper Rectal Cancer: An International Multi-Institutional Study
Source: Medicine (Baltimore). 2016 Jun 3;95(22):e2990. doi: 10.1097/MD.0000000000002990 (PMC4900695; doi:10.1097/MD.0000000000002990)
Supplement: Supplemental Digital Content [file medi-95-e2990-s001.docx]

Supplemental file 1: Statistical methods for Figure 2

In Figure 2, cumulative rate for local relapse according to tumor height (cm) was presented by follow steps; Step1. Calculate each recurrence rate by tumor height,
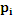

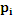
,
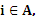

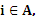

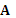

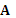
 is a finite set of tumor heights, Step2. Calculate sum of recurrence rates,
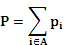

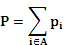
, Step3. Calculate each cumulative rate of recurrence,
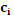

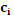
 i.e.
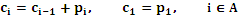

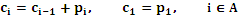
, Step4. Draw scatter plot X-axis as tumor height, Y-axis as
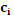

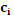
. However, recurrence rate may be affected not only tumor height but also other variables. Therefore, the effect of other variables was removed using propensity score matching (PSM) method with other variables as confounding (matching) variables. In PSM methods, logit was used for caliper indicating the maximum width of the caliper for which matching should be done, matching ratio was 18:1 and seed number is 35,417.

Supplemental Table 1. Patterns of Recurrence Following Primary Resection

| Site of Recurrence | Total set | | | | Subgroup set | | |
| --- | --- | --- | --- | --- | --- | --- | --- |
|  | Sigmoid  (n=678) | Upper rectum  (n=660) | Mid/Low rectum  (n=764) | *P* | Intreperitoneal  (n=858) | Extraperitoneal  (n=485) | *P* |
| Local site only, No.(%) | 12(1.8) | 18(2.7) | 83(10.8) | <0.001 | 40(4.6) | 64(13.2) | <0.001 |
| Lateral Pelvic^†^ | 4(0.6) | 8(1.2) | 56(7.3) | <0.001 | 22(2.5) | 45(9.2) | <0.001 |
| Central Pelvic^‡^ | 7(1.0) | 9(1.3) | 19(2.5) | 0.08 | 16(1.8) | 12(1.4) | 0.58 |
| Other site | 1(0.1) | 1(0.1) | 8(1.0) | 0.02 | 2(0.2) | 7(1.4) | 0.002 |
| Distant site only, No.(%) | 97(14.3) | 128(19.4) | 180(23.6) | <0.001 | 186(21.6) | 119(24.5) | 0.26 |
| Liver | 49(7.2) | 70(10.6) | 66(8.6) | 0.09 | 92(10.7) | 42(8.6) | 0.26 |
| Lung | 27(4.0) | 37(5.6) | 87(11.4) | <0.001 | 67(7.8) | 57(11.7) | 0.02 |
| Peritoneal cavity | 13(1.9) | 6(0.9) | 11(1.4) | 0.30 | 10(1.1) | 7(1.4) | 0.86 |
| Other site | 8(1.2) | 15(2.2) | 16(2.1) | 0.96 | 17(1.9) | 13(2.6) | 0.52 |
| Local and distant, No.(%) | 4(0.6) | 13(1.9) | 10(1.3) | 0.08 | 16(1.4) | 7(1.4) | 0.72 |

^†^lateral pelvic including pelvic side-wall lymph node-bearing areas along the obturator, the internal, external, common iliac vessels, and the pelvic nerve plexus

^‡^central pelvic site including recurrence in the tumor bed, anastomosis site, posterior and anterior pelvic organs (bladder, prostate, vagina, sacral promontory etc.), and perineum.

Supplemental Table 2. Pooled Rectal Cancer Analysis: Impact of TN stage on Local Relapse and Survival

|  |  | Sigmoid | Upper rectum | Mid / Low rectum | *P* |
| --- | --- | --- | --- | --- | --- |
| 5-year Local recurrence rate, %* | | | | | |
| T3N0 |  | 1.5(0-3.0) | 1.2(0-2.4) | 7.4(4.7-10.1) | <0.001  a,b < c† |
| T3N1-2 |  | 2.7(0.5-4.8) | 5.7(2.1-9.3) | 16.0(10.9-21.1) | <0.001  a,b < c† |
| T4N0 |  | 3.8(0-11.2) | 5.6(0-16.1) | 16.7(0-37.8) | 0.32 |
| T4N1-2 |  | 9.2(0.6-17.9) | 17.2(4.5-29.8) | 38.1(11.7-64.4) | 0.01  a <b< c† |
| 5-Year Disease-Free survival, %* | | | | | |
| T3N0 |  | 87.6(83.7-91.5) | 78.2(73.8-82.7) | 68.6(64.4-73.2) | <0.001  a>b,c† |
| T3N1-2 |  | 80.4(75.3-85.1) | 63.4(56.6-70.2) | 50.2(44.0-56.4) | <0.001  a > b > c† |
| T4N0 |  | 77.8(62.6-92.9) | 70.3(58.4-89.8) | 66.7(40-93.3) | 0.74 |
| T4N1-2 |  | 61.6(48.6-73.5) | 59.5(44.7-74.9) | 50.2(25.5-74.3) | 0.51 |
| 5-Year Overall Survival, %* | | | | | |
| T3N0 |  | 90.1(86.7-93.7) | 81.8(76.1-84.5) | 77.1(73.0-81.2) | <0.001  a>b,c† |
| T3N1-2 |  | 91.6(88.1-95.0) | 77.8(70.8-83.7) | 68.1(62.4-73.8) | <0.001  a>b,c† |
| T4N0 |  | 80.5(65.2-95.6) | 80.0(62.5-97.5) | 83.3(62.2-100) | 0.97 |
| T4N1-2 |  | 74.6(63.5-85.7) | 68.7(54.4-82.9) | 56.3(31.9-80.6) | 0.26 |

* Values are mean (95% confidence interval).

† Post hoc multiple comparison test.
